# Access to and Use of Internet and Social Media by Low-Morbidity Stroke Survivors Participating in a National Web-Based Secondary Stroke Prevention Trial: Cross-sectional Survey

**DOI:** 10.2196/33291

**Published:** 2022-05-30

**Authors:** Brigid Clancy, Billie Bonevski, Coralie English, Amanda L Baker, Alyna Turner, Parker Magin, Michael Pollack, Robin Callister, Ashleigh Guillaumier

**Affiliations:** 1 The University of Newcastle Callaghan Australia; 2 Hunter Medical Research Institute New Lambton Heights Australia; 3 Flinders University Bedford Park Australia; 4 Deakin University Geelong Australia; 5 Hunter New England Local Health District New Lambton Heights Australia

**Keywords:** stroke, stroke survivor, recurrent stroke, digital health, social media, internet use, eHealth, information-seeking behavior, web-based, mobile phone

## Abstract

**Background:**

eHealth applications for stroke are a growing area of research that has yielded promising results. However, little is known about how stroke survivors engage with the internet, social media, and other digital technologies on a day-to-day basis.

**Objective:**

This study had three main objectives: to describe the type, frequency, and purpose of technology use among a cohort of low-morbidity stroke survivors; to investigate associations between social media use and participant factors, including sociodemographics, physical function, and independence in activities of daily living; and to investigate associations between stroke-related health risk factors and the use of the internet to search for health and medical information.

**Methods:**

This study is a secondary analysis of data obtained during a national randomized controlled trial—*Prevent 2^nd^ Stroke*. The participants were stroke survivors recruited from 2 Australian stroke registries who completed 2 telephone-administered surveys to collect data on demographics and stroke characteristics; health risk factors (diet quality, physical activity, blood pressure medication, alcohol intake, anxiety and depression, and smoking status); physical functioning; independence in activities of daily living; and questions about what technology they had access to, how often they used it, and for what purposes. Participants were eligible if they had no more than a moderate level of disability (modified Rankin score ≤3) and had access to the internet. Multivariable logistic regression was used to assess the associations between social media use and sociodemographics, physical function, and independence in activities of daily living as well as associations between stroke-related health risk factors and the use of the internet to search for health and medical information.

**Results:**

Data from 354 participants were included in the analysis. Approximately 79.1% (280/354) of participants used the internet at least daily, 40.8% (118/289) accessed social media on their phone or tablet daily, and 46.4% (134/289) looked up health and medical information at least monthly. Women were 2.7 times more likely to use social media (adjusted odds ratio 2.65, 95% CI 1.51-4.72), and people aged >75 years were significantly less likely to use social media compared with those aged <55 years (adjusted odds ratio 0.17, 95% CI 0.07-0.44). Health risk factors were not found to be associated with searching for health- or medical-related information.

**Conclusions:**

The internet appears to be a viable platform to engage with stroke survivors who may not be high-morbidity to conduct research and provide information and health interventions. This is important given that they are at high risk of recurrent stroke regardless of their level of disability. Exploring the technology use behaviors and the possibility of eHealth among survivors who experience higher levels of morbidity or disability because of their stroke is an area of research that warrants further study.

## Introduction

### Background

Stroke is a leading cause of death and disability around the world [1]. Recurrent strokes are estimated to account for approximately one-quarter of all stroke incidents [2]. Data from the United States [3], United Kingdom [4], and Australia [5] suggest that stroke survivors do not receive adequate support and information on secondary stroke prevention following acute treatment. There is a need to develop better ways of reaching stroke survivors to involve them in health research as well as to deliver support and health information. Technology may be a part of this solution.

eHealth refers to health services or information that are delivered or enhanced through the use of the internet and related technologies [6]. eHealth has many potential applications in stroke prevention and care. Telerehabilitation has been one of the main areas of eHealth application in stroke and has been identified in multiple systematic reviews as a potential alternative or adjunct to usual rehabilitation [7-9], especially in the context of COVID-19 [10]. eHealth interventions aimed at reducing cardiovascular risk factors have shown promising results in glycemic control, achieving smoking cessation, diet and weight management, and increasing physical activity, and are generally acceptable and feasible [11-14]. A recent systematic review found that information and communication technology interventions aimed at stroke survivors and their carers are likely to provide some benefit, although the heterogeneity in study design and outcomes measured makes it difficult to draw nuanced conclusions [15].

Digital tools used in research studies, such as social media, data mining, email, and SMS text messaging, may be effective ways to engage stroke survivors in research. Digital tools are increasing in popularity for recruiting and retaining participants in randomized controlled trials, with the number of published studies doubling in the past decade [16]. Social media is a type of technology that is increasingly being used as a recruitment tool [17]. Little is known about how stroke survivors, who may be experiencing cognitive or physical disabilities, engage with social media and other technologies. Limitations on typing as well as a tendency for others’ attention to focus on a person’s disability have been associated with negative experiences for people with physical disabilities using social media [18].

The percentage of Australians using the internet to access health services has more than doubled from 22% in 2014-2015 to 46% in 2016-2017 [19]. Approximately 40% of Australians aged >55 years have accessed the internet for this purpose [19]. A study from the United States found that 57% of patients with acute coronary syndrome who had accessed the internet in the past 4 weeks reported web-based health information seeking [20]. However, there are no data available on web-based health and medical information seeking in people who have experienced stroke. A previous study on the web-based health information–seeking behaviors of people with chronic disease found that the third most common thing people searched for was lifestyle information such as diet and exercise [21]. It is possible that people who have experienced stroke are also looking to the internet for lifestyle information about health risk factors related to recurrent stroke, such as physical activity, diet, alcohol use, smoking, anxiety, and depression [22-24].

Despite the increasing popularity of eHealth use in this group, little is known about how stroke survivors engage with technology. A recent cross-sectional survey of stroke survivors (n=248) and carers (n=127) in the United States found that 81% of stroke survivors and 97% of carers had internet access and that smartphones were the most common device used to access the internet [25]. Beyond this, although information and communication technology interventions aimed at stroke survivors and their carers are likely to provide benefits, there is little information available about how stroke survivors use technology in their day-to-day lives [15]. A better understanding of this general use would assist in understanding how many people with lived experience of stroke have the potential to engage with eHealth programs or research.

### Objectives

This study examined how a sample of Australian survivors of stroke who participated in a web-based secondary stroke prevention trial used technology. This study had three main objectives: (1) to describe the type, frequency, and purpose of technology use among a cohort of survivors of stroke; (2) to investigate associations between social media use and participant factors, including sociodemographics, physical function, and independence in activities of daily living; and (3) to investigate associations between stroke-related health risk factors (physical activity, diet, alcohol intake, smoking, and psychological distress) and using the internet to search for health and medical information.

## Methods

### Study Design

This study is a secondary analysis of cross-sectional data obtained during a national randomized controlled trial—*Prevent 2^nd^ Stroke* [26,27]. Details regarding the study design and methods have been published elsewhere [26]. In short, trial participants (N=399) were recruited from the Australia-wide Australian Stroke Clinical Registry (AuSCR) and the Hunter Stroke Research Volunteer Registry (HSRVR), which recruits stroke survivors from the Hunter region of New South Wales.

The baseline survey was administered via a computer-assisted telephone interview and included questions about participant demographics, stroke characteristics, health risk behaviors, and mood. The participants were then randomly allocated (1:1) to the intervention group, who received 12 weeks of access to the *Prevent 2^nd^ Stroke* web-based program, or to the control group, who received internet addresses of readily available, generic web-based health programs designed for the general population. Six months after the initial baseline survey, a follow-up computer-assisted telephone interview survey was conducted (n=356), which included questions about the use of computers, tablets, phones, and the internet.

### Ethics Approval

Ethical approval was obtained through the University of Newcastle Human Research Ethics Committee (H-2017-0051).

### Participants

The AuSCR and HSRVR each used the eligibility criteria to screen their registrant databases and sent invitation packs on behalf of the study team to potentially eligible individuals. Individuals were eligible to take part in the study if they were aged ≥18 years, were part of the AuSCR or HSRVR, had had their most recent stroke in the previous 6-36 months, were fluent in English, and had access to the internet via a home device (eg, computer, tablet, or smartphone) or were willing to use public internet services (eg, public library). The participants were required to be able to walk without assistance and have no more than a moderate disability and so were ineligible if they scored ≥4 on the modified Rankin Scale [28].

### Measures

Some measures were taken during the baseline survey either because they were static measures or to reduce the chance of health risk factor measures being affected by the intervention. The following measures were taken at the baseline survey.

#### Demographic and Stroke Characteristics

Age (<55, 55-64, 65-74, and >74 years), sex (male or female), weekly personal gross income (low: <Aus $399 [US $291.72], mid: Aus $400-$999 [US $292.45-$730.39], and high: ≥Aus $1000 [US $731.12]), whether they identify as Aboriginal or Torres Strait Islander or both, country of birth (Australia or other), stroke type (stroke or transient ischemic attack), and yes or no to whether it was their first stroke event.

#### Diet Quality

The Australian Recommended Food Score questionnaire was administered to assess usual diet quality, and scores were categorized as *needs work* (<33), *getting there* (33-38), *excellent* (39-46), or *outstanding* (≥47) [29].

#### Physical Activity

The Godin Leisure-Time Exercise Questionnaire was used to assess physical activity levels. The participants’ responses were scored as *active* (≥24), *moderately active* (14-23), or *sedentary* (0-13) [30].

#### Blood Pressure Medication

Respondents were asked *Are you on blood pressure medications?* with response options *yes*, *no*, and *don’t know*.

#### Alcohol Intake

The Alcohol Use Disorders Identification Test–Consumption was used to assess alcohol intake, and respondents with a score of ≥3 in women or ≥4 in men were considered to be drinking at potentially risky levels [31].

#### Psychological Distress, Anxiety, and Depression

The Patient Health Questionnaire–4 is an ultrabrief screening scale used as a measure of psychological distress and to screen for anxiety and depression [32]. Psychological distress was measured using all 4 questions and a score range of 0-12, with higher scores indicating greater psychological distress. Anxiety was measured using items 1 and 2, and depression was measured using items 3 and 4. A score of ≥3 for either pair of items was considered positive for anxiety or depression, respectively.

#### Smoking Status

Respondents were asked *do you currently smoke tobacco products?* with response options being *yes, daily*; *yes, once a week*; *yes, less than once a week*; and *no* [33]. This was used to determine the current smoking status of the participants.

#### Physical Functioning and Independent Living

The Barthel Index was used to assess physical functioning [34,35], where a score of 100 was considered *independent*, a score of 91-99 was considered *slight dependence*, and a score of 61-90 was considered *moderate dependence*. The Instrumental Activities of Daily Living was used to assess independent living ability [36], where a score of 8 was considered *independent living*, a score of 7 was considered *mostly independent living*, and a score of 0 to 6 was considered *requiring assistance*.

The technology-related measures were taken during the follow-up survey as this was the only stage at which they were asked. The potential effects of the intervention on these responses are discussed in the *Data Analysis* section. The participants were asked about what technology they had access to (including if it was internet-enabled); frequency of internet use; and how often they used their mobile phone or tablet for different purposes such as sending and receiving calls and SMS text messages, social media use, and accessing health- and medical-related information. These measures were adapted from the Australian Rural Mental Health Study [37] and the Pew Internet & American Life Poll 2012 Health Tracking Survey [38]. The survey questions used are presented in Multimedia Appendix 1.

### Data Analysis

Descriptive statistics were used to describe the participants’ access to technology, frequency of internet use, and purpose and frequency of use of a mobile phone or tablet. This included frequencies with percentages of nonmissing observations for categorical variables and mean with SD values for continuous variables.

Logistic regression modeling was used to identify associations between social media use and stroke survivor demographics, psychological distress, physical functioning, and independent living. Logistic regression modeling was also used to identify whether the presence of health risk factors was associated with the use of a mobile phone or tablet to look for health- or medical-related information. Adjusted odds ratios (AORs) with 95% CIs and *P* values are provided. *P*<.05 was considered statistically significant.

The items regarding technology use were asked after the sample had participated in a trial encouraging technology use. To assess whether this affected the results (ie, overestimating typical use), logistic regression modeling was used to identify associations between group allocation and answers to the technology-related questions. No significant difference between groups in terms of how they used their mobile phone or tablet was found. There was a significant (*P*=.02) association between allocation and frequent use of internet (ie, once a week or more), with frequent internet use being 3.1 (95% CI 1.2-8.0) times more likely for people allocated to the intervention group than to the control group. However, because of the low number of participants who were infrequent internet users (25/354, 7.1%) and the fact that it did not appear to affect the responses used in the other logistic regressions on social media use and health and medical information, we determined that it was more appropriate to retain the larger sample size for this study than to exclude those in the intervention group.

Statistical analyses were programmed using R (version 4.0.2; R Foundation for Statistical Computing) [39].

## Results

### Participant Characteristics

Of the original 399 trial participants, 2 (0.5%) did not fully complete the follow-up survey and missed the technology-related questions, 38 (9.5%) were unable to be contacted for the follow-up survey, and 5 (1.3%) withdrew from the trial for personal reasons relating to their own health or care for others. In total, 354 participants completed the follow-up survey (333/354, 94.1% from the AuSCR and 21/354, 5.9% from the HSRVR). Most participants were male (231/354, 65.3%), and their average age was 68 (SD 12) years. Most participants were born in Australia (272/354, 76.8%). Table 1 shows the characteristics of the 354 participants who completed the follow-up survey.

**Table 1:** Sociodemographic and health risk factor characteristics of the participants (N=354).

Characteristic		Values^a^
Age (years), mean (SD)		68 (12)
**Age categories (years), n (%)**		
	<55	41 (11.6)
	55-64	71 (20.1)
	65-74	136 (38.4)
	>74	105 (29.7)
Men, n (%)		231 (65.2)
**Income (Aus $; US $), n (%)**		
	Low (<399; <291.72)	97 (27.4)
	Mid (400-999; 292.45-730.39)	139 (39.3)
	High (≥1000; ≥731.12)	87 (24.6)
	Do not know or did not answer	30 (8.5)
Aboriginal or Torres Strait Islander or both (yes), n (%)		1 (0.3)
Country of birth (Australia), n (%)		272 (76.8)
**Stroke type, n (%)**		
	Stroke	216 (61)
	TIA^b^	125 (35.3)
	Do not know	13 (3.7)
**History of previous stroke, n (%)**		
	No, first episode	328 (92.7)
	Yes, had TIA before	15 (4.2)
	Yes, had stroke before	5 (1.4)
	Do not know	6 (1.7)
**Diet quality (ARFS^c^ score), n (%)**		
	Needs work (<33)	77 (21.8)
	Getting there (33-38)	65 (18.4)
	Excellent (39-46)	152 (42.9)
	Outstanding (≥47)	101 (28.5)
**Physical activity (GLTEQ^d^ score), n (%)**		
	Sedentary (0-13)	116 (32.8)
	Moderately active (14-25)	101 (28.5)
	Active (≥24)	179 (50.6)
**Blood pressure medication, n (%)**		
	No	111 (31.4)
	Yes	275 (77.7)
	Do not know	10 (2.8)
**Potentially risky drinking (AUDIT-C^e^), n (%)**		
	No	207 (58.5)
	Yes	189 (53.4)
**Anxiety (PHQ-4^f^ score), n (%)**		
	Low (0-2)	348 (98.3)
	Anxiety (3-6)	46 (13)
**Depression (PHQ-4 score), n (%)**		
	Low (0-2)	347 (98)
	Depression (3-6)	46 (13)
Current smoker (yes), n (%)		16 (4.5)
**Physical functioning (Barthel Index), n (%)**		
	Independent (100)	298 (84.2)
	Slight dependence (91-99)	71 (20.1)
	Moderate dependence (61-90)	24 (6.8)
**Independent living (IADL^g^ score), n (%)**		
	Independent living (8)	326 (92.1)
	Mostly independent living (7)	39 (11)
	Requiring assistance (0-6)	31 (8.8)

^a^Not all n values add up to 354 because of missing data.

^b^TIA: transient ischemic attack.

^c^ARFS: Australian Recommended Food Score.

^d^GLTEQ: Godin Leisure-Time Exercise Questionnaire.

^e^AUDIT-C: Alcohol Use Disorders Identification Test–Consumption.

^f^PHQ-4: Patient Health Questionnaire–4.

^g^IADL: Instrumental Activities of Daily Living.

### Type, Frequency, and Purpose of Technology Use

Table 2 shows the participants’ access to technology. Most participants (319/354, 90.1%) had access to a computer with internet access, and 73.4% (260/354) had access to a mobile phone with internet access.

Table 3 shows the frequency of internet use among the whole sample of participants, with most accessing the internet at least daily (280/354, 79.1%) and only 4.8% (17/354) answering that they did not use the internet.

Table 4 shows the purpose and frequency of use of the participants who answered *yes* to having access to a mobile phone or tablet with internet access in Table 2 (289/354, 81.6%). Half of these participants used their mobile phone or tablet to access social media networking sites at least weekly (149/289, 51.6%), whereas 46.4% (134/289) looked for health- or medical-related information at least monthly. Mobile phones and tablets were used daily for communication purposes, including phone calls (234/289, 81%), text messages (203/289, 70.2%), and emails (139/289, 48.1%).

**Table 2:** Access to technology at home or elsewhere (N=354).

Access to technology	Yes, n (%)
Computer with internet access	319 (90.1)
Mobile phone with internet access	260 (73.4)
Tablet device with internet access	209 (59)
Webcam	113 (31.9)
Mobile phone without internet access	73 (20.6)
Tablet device without internet access	13 (3.7)
Computer without internet access	5 (1.4)
None of the above	2 (0.6)

**Table 3:** Frequency of internet use, including email (N=354).

Frequency of internet use	Total, n (%)
Several times a day	174 (49.2)
Every day	106 (29.9)
Several times a week	33 (9.3)
Once a week	16 (4.5)
Once a month or less	7 (2)
I do not use the internet	17 (4.8)
Do not know	1 (0.3)

**Table 4:** Purpose and frequency of use of mobile phone or tablet (N=289).

	Never, n (%)	Less than monthly, n (%)	Monthly, n (%)	Weekly, n (%)	Daily, n (%)	Do not know, n (%)
Making or receiving phone calls	6 (2.1)	4 (1.4)	12 (4.2)	32 (11.1)	234 (81)	1 (0.3)
Sending or receiving SMS text messages	23 (8)	6 (2.1)	4 (1.4)	52 (18)	203 (70.2)	1 (0.3)
Accessing the internet	36 (12.5)	7 (2.4)	6 (2.1)	43 (14.9)	196 (67.8)	1 (0.3)
Using apps	55 (19)	12 (4.2)	14 (4.8)	36 (12.5)	169 (58.5)	3 (1)
Accessing social media networking sites (eg, Facebook, Twitter, or Instagram)	127 (43.9)	8 (2.8)	5 (1.7)	31 (10.7)	118 (40.8)	0 (0)
Sending or receiving emails	61 (21.1)	13 (4.5)	13 (4.5)	62 (21.5)	139 (48.1)	1 (0.3)
Taking pictures (photos)	40 (13.8)	30 (10.4)	66 (22.8)	95 (32.9)	58 (20.1)	0 (0)
Looking for health- or medical-related information	112 (38.8)	42 (14.5)	63 (21.8)	53 (18.3)	18 (6.2)	1 (0.3)
For entertainment (eg, music, watching videos, or Netflix)	138 (47.8)	15 (5.2)	20 (6.9)	45 (15.6)	70 (24.2)	1 (0.3)

### Associations Between Participant Characteristics and Social Media Use

Table 5 shows the multivariable regression results for outcome use of phone or tablet to access social media networking sites. It includes only the participants who answered *yes* to having access to a mobile phone or tablet with internet access in Table 2 (289/354, 81.6%). After accounting for the other variables in the model, age and sex showed a statistically significant association with social media use, with younger persons and women more likely to use social media. Women were 2.7 times more likely to use social media compared with men (AOR 2.65, 95% CI 1.51-4.72). Being aged >55 years was associated with lower odds of using social media compared with those aged <55 years; however, only the highest age category (≥75 years) was significantly different from <55 years (AOR 0.17, 95% CI 0.07-0.44). None of the other variables assessed showed a statistically significant association.

**Table 5:** Multivariable regression results for outcome use of phone or tablet to access social media networking sites (N=289).

Variable		Use of social media via phone or tablet, n (%)			Multivariable	
		No	Yes	OR^a^ (95% CI)		*P* value
**Age categories (years)**		135 (100)	153 (100)			.001
	<55	10 (7.4)	31 (20.3)	—^b^		
	55-64	29 (21.5)	35 (22.9)	0.46 (0.18-1.11)		
	65-74	52 (38.5)	68 (44.4)	0.50 (0.21-1.15)		
	>74	44 (32.6)	19 (12.4)	0.17 (0.07-0.44)		
**Sex**		135 (100)	154 (100)			.001
	Male	100 (74.1)	83 (53.9)	—		
	Female	35 (25.9)	71 (46.1)	2.65 (1.51-4.72)		
**Income (Aus $; US $)**		135 (100)	154 (100)			.83
	Low (<399; <291.72)	38 (28.1)	44 (28.6)	—		
	Mid (400-999; 292.45-730.39)	52 (38.5)	54 (35.1)	1.03 (0.54-1.96)		
	High (>999; >730.39)	33 (24.4)	46 (29.9)	1.29 (0.61-2.74)		
	Do not know or refused	12 (8.9)	10 (6.5)	0.80 (0.29-2.21)		
**Psychological distress (PHQ-4^c^ score)**		134 (100)	153 (100)			.87
	None (0-2)	100 (74.6)	116 (75.8)	—		
	Mild (3-5)	24 (17.9)	24 (15.7)	0.84 (0.42-1.68)		
	Moderate or severe (6-12)	10 (7.5)	13 (8.5)	1.04 (0.40-2.76)		
**Physical functioning (Barthel Index)**		135 (100)	154 (100)			.83
	Independent (100)	104 (77)	120 (77.9)	—		
	Slight dependence (91-99)	24 (17.8)	26 (16.9)	0.83 (0.40-1.71)		
	Moderate dependence (61-90)	7 (5.2)	8 (5.2)	0.75 (0.21-2.68)		
**Independent living (IADL^d^ score)**		135 (100)	154 (100)			.91
	Independent living (8)	117 (86.7)	134 (87)	—		
	Mostly independent living (7)	10 (7.4)	10 (6.5)	1.16 (0.42-3.21)		
	Requiring assistance (0-6)	8 (5.9)	10 (6.5)	1.25 (0.39-4.17)		

^a^OR: odds ratio.

^b^There was no comparison in these cells.

^c^PHQ-4: Patient Health Questionnaire–4.

^d^IADL: Instrumental Activities of Daily Living.

### Associations Between Health Risk Factors and Health Information Searching

In the multivariable regression conducted to determine whether the use of a phone or tablet to find medical information was related to the presence of health risk factors, no results were significant. None of the health risk factor variables were associated with searching for medical information on the web on a phone or tablet.

## Discussion

### Principal Findings

This study found that most participants owned a computer, mobile phone, or tablet with internet access and accessed the internet at least daily. Internet access was a requirement for participation; therefore, these are unsurprising results for this particular cohort. Daily activities on these devices for most of the sample included making or receiving phone calls, sending or receiving SMS text messages, accessing the internet, or using apps. Most also accessed social media at least weekly (149/289, 51.6%), and almost half of them accessed health- or medical-related information at least monthly (134/289, 46.4%).

We found that stroke survivors, including those in older age groups, frequently use the internet for a number of purposes. A previous US study of stroke survivors with a similar average age (64 years) reported that 81% had access to the internet, with most accessing it for >5 hours per week [25], whereas a Danish study of 100 people who were patients in a stroke unit found that 87% reported having access to an internet-enabled device at home [40]. In 2016-2017 in Australia, 55% of the general population aged >65 years had used the internet in the last 3 months, an increase on the 46% from 2012 to 2013, and two-thirds had accessed it for health services [19]. This shows that, although internet use among older people may be lower than among the rest of the population, there is still a significant proportion of older people engaging with it, and this use is on the rise. The increasing rates of stroke among younger adults cannot be ignored when examining technology use among stroke survivors. Hospitalization rates for acute ischemic stroke have increased significantly between 2003 and 2012 among men (41.5%) and women (30%) aged between 35 and 44 years [41]. With the rate of internet access among this group at 96% [19], it is important to consider this younger age group in the development of internet-based interventions. It can be inferred that, with the increasing number of young people experiencing stroke, in combination with the increasing use of the internet by older age groups and the already significant use of the internet in the current stroke survivor population, digital literacy among those who have experienced stroke will only become more prevalent. As digital literacy increases and more health and medical information and interventional programs are available on the web, a targeted effort is needed to ensure that stroke survivors who are interested in these sorts of programs, including those who face communication difficulties, are not left behind.

Previous research has found that stroke-related information provision for people with lived experience of stroke is insufficient [42,43]. More than 50% of stroke survivors have self-reported an unmet need for stroke information [4]. In Australia, only 63% of patients in inpatient stroke rehabilitation services receive education about stroke, lifestyle management, secondary stroke prevention, and recovery [5]. With many stroke survivors having access to the internet, it is not unreasonable to assume that some are looking on the web to fill these information gaps. Approximately 46.4% (134/289) of our sample used their mobile phones or tablet devices to look up health or medical information at least once a month. This is congruent with previous research that found that 57% of patients with acute coronary syndrome who had accessed the internet in the past 4 weeks had used it for web-based health information seeking [20]. The quality and accessibility of the information available to stroke survivors on websites are mixed [44], and much of this information does not meet the recommended readability guidelines for stroke survivors [45]. Some people who have experienced stroke also turn to unregulated options such as web-based forums for information [46]. With many stroke survivors lacking adequate stroke-related information and turning to the internet for additional health and medical information, there is a need to ensure that the information available on the web is not only accurate and appropriate but also accessible for this population.

Although digital health presents opportunities for greater reach to promote health, it is not a one-size-fits-all solution. It is likely to be most suited to those with lower morbidity or disability, with people who are more greatly affected by stroke requiring more intensive resources [47]. Nonetheless, all stroke survivors are at higher risk of recurrent stroke than the general population [48], and all avenues should be explored to reduce their risk of recurrent stroke. This study suggests that there is an opportunity to use digital health applications to reduce the risk of recurrent stroke among survivors with low levels of disability. Further studies will be required to explore this application in people who experience greater morbidity and disability as a result of stroke.

Among our sample of low-morbidity stroke survivors, we investigated whether there was a relationship between the presence of health risk factors (diet quality, physical activity, blood pressure medication, alcohol intake, anxiety and depression, and smoking status) and using the internet to search for health-related information. However, we did not find any associations. This may be due to a number of reasons, such as the survey questions used not being specific enough; individuals not being aware of their risk or not seeing their behavior as problematic; or participants obtaining that type of information elsewhere, such as a managing general practitioner. However, most of this sample looked for health information on the web regardless of their health risk factors. This presents a clear opportunity to provide another mode of education and engagement around health risk factors and risk of recurrent stroke in a population that often misses out on receiving secondary stroke prevention education [4,5].

Social media platforms are also of increasing interest to health researchers [49]. They offer a low or no-cost means of observing and reaching both diverse and narrow audiences with the possibility of multidirectional communication [49]. Social media was identified as an effective recruitment tool in a randomized controlled trial for hypertension [50]. In the general Australian population, 51% of people aged >65 years who had accessed the internet in the last 3 months had accessed social media during this time [19]. We had similar findings within our sample, with just over half of the participants (149/289, 51.6%) accessing social media sites at least weekly, whereas 43.9% (127/289) did not access them at all. On the basis of our results, researchers looking to use social media to observe or access stroke survivor populations may face more difficulty accessing male stroke survivors and those aged ≥75 years. Social media may be a feasible platform for recruiting some groups of stroke survivors.

### Strengths and Limitations

This study provides data from a large national sample of stroke survivors. The participants in this study were technology users by nature given their recruitment to a web-based secondary stroke prevention trial where the use of an internet-enabled device and email were required. They were also a relatively *well* cohort compared with the general stroke population, with high levels of independence and limited disability because of the eligibility requirement of a modified Rankin score of ≤3. Unfortunately, this means that the trial excluded many of those who are significantly affected by their stroke-related impairments and cannot be generalized to the Australian stroke survivor population as a whole. More research into the internet use behaviors of those with greater levels of disability and dependence, as well as how specific impairments may affect the use of the internet, is warranted. However, this study does provide an indication of the patterns of technology use among more able stroke survivors who are already internet users and willing to participate in internet-based research. This population still requires support in managing their health risk factors, and this research is beneficial for better understanding how to access them and deliver appropriate care.

The questions assessing social media use, accessing health- and medical-related information, and other device-related activities were also only asked in the context of mobile phone and tablet use. The questions did not account for alternative modes of internet access such as a desktop or laptop computer, which may lead to an underrepresentation of stroke survivor engagement with these activities.

### Conclusions

The internet may be a viable platform to engage with stroke survivors experiencing low levels of disability for health interventions, information, and research. This is important as all stroke survivors are at higher risk of stroke than the general population and require secondary stroke prevention support and education. Exploring the technology use behaviors and possibility of eHealth with people who experience greater levels of disability following stroke is an area of research that warrants further study.

## Appendix

Multimedia Appendix 1 Technology use survey questions.

## Abbreviations

AOR: adjusted odds ratio
AuSCR: Australian Stroke Clinical Registry
HSRVR: Hunter Stroke Research Volunteer Registry
