# Supplementary material for: Access to and Use of Internet and Social Media by Low-Morbidity Stroke Survivors Participating in a National Web-Based Secondary Stroke Prevention Trial: Cross-sectional Survey
Source: J Med Internet Res. 2022 May 30;24(5):e33291. doi: 10.2196/33291 (PMC9153916; doi:10.2196/33291)
Supplement: Multimedia Appendix 1 [file jmir_v24i5e33291_app1.docx]

**MULTIMEDIA APPENDIX 1**

Technology use survey questions.

1) Do you have easy access to any of the following, at home or elsewhere?

| a. Computer without internet access | Yes | No |
| --- | --- | --- |
| b. Computer with internet access | Yes | No |
| c. Mobile phone without internet access | Yes | No |
| d. Mobile phone with internet access | Yes | No |
| e. Tablet device without internet access | Yes | No |
| f. Tablet device with internet access | Yes | No |
| g. Webcam | Yes | No |

2) How often do you use/access the internet (including email)?

| I do not use the internet | 1 |
| --- | --- |
| Once a month or less | 2 |
| Once a week | 3 |
| Several times a week | 4 |
| Every day | 5 |
| Several times a day | 6 |

*If Q1 D or F = ‘Yes’ present Q3*

3) How often do you use your [smartphone / tablet] to do any of the following things?

|  | Never | Less than once a month | Once or twice a month | Weekly | Daily |
| --- | --- | --- | --- | --- | --- |
| Make or receive phone calls | 1 | 2 | 3 | 4 | 5 |
| Send or receive text messages (SMS) | 1 | 2 | 3 | 4 | 5 |
| Access the internet | 1 | 2 | 3 | 4 | 5 |
| Use applications (‘apps’) | 1 | 2 | 3 | 4 | 5 |
| Social networking (e.g. Facebook, Twitter) | 1 | 2 | 3 | 4 | 5 |
| Send or receive email | 1 | 2 | 3 | 4 | 5 |
| Take a picture | 1 | 2 | 3 | 4 | 5 |
| Look for health or medical information | 1 | 2 | 3 | 4 | 5 |
| Entertainment (listen to music, watch videos) | 1 | 2 | 3 | 4 | 5 |
